# Is the Urine Cannabinoid Level Measured via a Commercial Point-of-Care Semiquantitative Immunoassay a Cannabis Withdrawal Syndrome Severity Predictor?

**DOI:** 10.3389/fpsyt.2020.598150

**Published:** 2020-12-03

**Authors:** Benedikt Bernd Claus, Michael Specka, Heath McAnally, Norbert Scherbaum, Fabrizio Schifano, Udo Bonnet

**Affiliations:** ^1^Department of Psychiatry, Psychotherapy and Psychosomatic Medicine, Evangelisches Krankenhaus Castrop-Rauxel, Academic Teaching Hospital of the University of Duisburg-Essen, Castrop-Rauxel, Germany; ^2^Department of Psychiatry and Psychotherapy, Faculty of Medicine, LVR-Hospital Essen, University of Duisburg-Essen, Essen, Germany; ^3^Department of Anesthesiology and Pain Medicine, University of Washington School of Medicine, Seattle, WA, United States; ^4^Psychopharmacology, Drug Misuse and Novel Psychoactive Substances Research Unit, School of Life and Medical Sciences, University of Hertfordshire, Hatfield, United Kingdom

**Keywords:** urinary 11-nor-9-carboxy-delta9-tetrahydrocannabinol, gender effect, cannabis withdrawal syndrome subtypes, protracted withdrawal syndrome, inpatient detoxification treatment

## Abstract

**Background:** For cannabis-dependent subjects, the relationship between cannabis withdrawal syndrome (CWS) severity and the urine cannabinoid concentrations are unclear; we investigated this using a commercial point-of-care (POC) enzyme immunoassay detecting 11-nor-9-carboxy-Delta-9-tetrahydrocannabinol (THC-COOH).

**Methods:** Observational study of 78 adult chronic cannabis-dependent subjects assessed over a 24-day inpatient detoxification treatment, with 13 serial measurement days. Repeated Measures Correlation and Multilevel Linear Models were employed.

**Results:** Absolute urinary THC-COOH levels significantly correlated with Marijuana Withdrawal Checklist (MWC) scores across the entire study duration (*r* = 0.248; *p* < 0.001). Correlation between serial creatinine-adjusted THC-COOH ratios and serial MWC scores emerged as significant only in the sample with higher MWC scores (>11 points) at admission (*n* = 21; *r* = 0.247; *p* = 0.002). The aforementioned significant relationships have persisted when replacing the absolute THC-COOH-levels with the (relative) day-to-day change in urinary THC-COOH levels. MWC scores were significantly correlated with the Clinical Global Impression-Severity (CGI-S; *r* = 0.812; *p* < 0.001). Females showed a significantly slower decline in urine THC-COOH levels and prolonged CWS course characterized by substantial illness severity (per CGI-S), occurring in nearly 30% of cases.

**Conclusion:** Urine cannabinoid levels (THC-COOH) determined by POC assay significantly predicted CWS severity (moderate correlation), guiding detoxification treatment duration. In patients with MWC > 11 points upon admission, creatinine-adjusted THC-COOH ratios also significantly predicted CWS severity—again with moderate effect size. Females showed prolonged urinary THC-COOH elimination and cannabis withdrawal.

## Introduction

The abrupt cessation of frequent cannabis intake is followed by a cannabis withdrawal syndrome (CWS), primarily presenting with emotional and behavioral symptoms (1–3). In US adults frequently using cannabis, the prevalence of CWS was 12.1% (4). Moreover, CWS is a key component of the cannabis dependence syndrome (CDS) as defined in ICD-10, with nearly 90% of these individuals displaying clinically relevant CWS (5). The cannabinoid receptor type 1 (CB_1_) is thought to play a major role in CWS occurrence (3, 6, 7). Discontinuation of synthetic cannabinoids (which are generally full CB_1_ agonists) (6) leads to a similar withdrawal syndrome (3); conversely, CB_1_ agonists alleviate CWS symptoms (3, 7).

Over the past 20 years, clinical characteristics of CWS have been described in many out- and inpatient as well as epidemiologic studies (1, 3). Operationalized CWS criteria were first provided in DSM-5 (8) and await revision and expansion in ICD-11 (3), with the magnitude of CWS severity generally associated with the extent and duration of cannabis use before quitting (1, 3, 7). The severity of CWS in heavy users is comparable with the burden of a moderate major depressive episode, a moderate alcohol withdrawal syndrome (9) or tobacco withdrawal (10), at times requiring in- (9) or outpatient (10) treatment.

The main psychotropic agent of natural cannabis is delta-9-tetrahydrocannabinol (THC) which is a CB_1_ partial agonist (3, 6). Main metabolites of THC include the psychotropic, water-soluble 11-hydroxy-delta-9-tetrahydrocannabinol (THC-OH), and the nonpsychotropic and lipophilic 11-nor-9-carboxy-delta-9-tetrahydrocannabinol (THC-COOH) (11). THC-COOH undergoes conjugation with glucuronic acid prior to excretion in the urine (12, 13) where it serves as a biomarker of cannabis use in commercial point-of-care drug screening tests (14, 15). However, urine THC-COOH levels alone cannot be used to determine either the timeframe or the amount of the last cannabis use (11, 16, 17). In Germany, where inpatient detoxification of cannabis users undergoing significant levels of CWS is supported by statutory health insurance (3), the termination of the inpatient detoxification treatment phase is often empirically determined by the observation of consistent urine THC-COOH levels below a cutoff point of 50 ng/ml (the sensitivity limit of most immunoassays) (14) which corresponds to the US federally mandated immunoassay cutoff concentration (18). However, it is unclear whether levels of this biomarker—as measured by point-of-care testing (POCT)—are really associated with CWS severity in clinical practice, which influences treatment decisions regarding discharge and subsequent outpatient rehabilitation treatment for CDS (3). Greater-severity CWS is not only associated with increased likelihood of CDS but also with increased comorbidity and negative psychosocial outcomes (1–4). The answer to the question of whether an easily determined POCT biomarker predicts CWS severity thus assumes greater importance in the context of resource allocation.

Toward that end, we investigated the correlation between the clinical CWS course of cannabis-dependent persons seeking inpatient detoxification treatment and the trends of their urine cannabinoid levels as measured by the semiquantitative DRI® Cannabinoid Assay (15). This inpatient environment allowed for good control of major potential confounding factors such as cannabinoid relapse, concomitant hidden drug or alcohol use, and environmental psychosocial stress and comorbidities (9). We also examined the potential confounding issues of intentional dilution and adipose tissue cannabinoid redistribution by assaying for both creatinine- and BMI-normalized urine cannabinoid levels (11–13).

## Methods

### Study Design, Participants, and Eligibility Criteria

This prospective observational study was conducted from 2008 to 2014 in an inpatient unit for detoxification from alcohol, prescription, and recreational drug abuse including cannabis at the Psychiatric University Hospital in Essen (LVR-Klinikum Essen), Germany. Adult detoxification-seeking patients who had a cannabinoid-positive urine screen upon admission were eligible. Only those patients who (a) were older than 18 years, (b) were diagnosed with cannabis dependence according to ICD-10 (19), (c) had used cannabis by inhalation daily or near-daily during the 6 months before admission, (d) reported use of cannabis within the 24 h prior to admission, (e) had used no other psychotropic substances (apart from tobacco) during the 4 weeks prior to admission, (f) presented with no active comorbid psychiatric or somatic disorder requiring treatment, (g) were familiar with the German language, and (h) gave their written informed consent were retained in the study. Inpatient treatment was scheduled for up to 24 days; however, patients could be discharged earlier based upon a shared patient/staff decision, when both parties agreed that the individual's psychiatric and somatic condition had improved to the point that primary or secondary care would be sufficient for continuation of treatment. In some cases, after inpatient stabilization, patients were referred to rehabilitation clinics for further treatment and support.

### Exclusion Criteria

Patients with documented (e.g., through breath analysis or urinalysis) relapse to use of cannabis or other substances including alcohol were excluded from the study. If there was reasonable suspicion of undisclosed Z-drugs (e.g., zolpidem, zaleplon, or zopiclone) or new psychoactive drugs (20), special urinalysis or serologic assays were performed and/or sent for detailed or confirmatory analysis to MVZ Synlab Leverkusen GmbH, Leverkusen, Germany, or the Division of Forensic Medicine at the University of Duisburg-Essen, Germany.

A ratio of 1.5 or greater between two serial creatinine-normalized urine THC-COOH values was interpreted as indication of relapse to marijuana use (21). In such cases, as previously described (9), blood THC-OH concentrations were assessed by gas chromatography-mass spectroscopy (GC-MS) for confirmation, and when an increase over admission baseline THC-OH was confirmed, the patient was excluded from the study on the basis of apparent cannabis relapse (9).

### Dropout Criteria

Dropouts included (a) premature self-discontinuation of treatment, (b) withdrawal of study participation consent, or (c) development of a relevant comorbidity requiring intervention and stabilization.

### Treatment Regimen, Including Medication-on-Demand

The multimodal inpatient treatment program consisted of a diverse regimen including regular medical assessments, individual and group psychotherapeutic sessions based upon motivational enhancement, cognitive-behavioral treatment elements and psychoeducation, physical and occupational therapies, and social counseling. In addition, the option for postdischarge transfer to a long-term rehabilitation program was offered to all patients. When patients showed distressing withdrawal symptoms such as anxiety, dysphoria, restlessness, or sleep disturbance, the nursing staff was allowed to administer escalating doses of gabapentin (22) (up to 600 mg q.i.d) or chlorprothixene (up to 50 mg q.i.d.) as medication-on-demand (PRN). For potential subanalysis purposes, an equipotency ratio assuming 50 mg chlorprothixene equivalent to 400 mg gabapentin was used.

### Measurements

Upon admission to detoxification treatment, a structured interview was administered to all patients to determine sociodemographics, addiction-related information (e.g., age at first cannabis use, amount and duration of daily cannabis use, other comorbid substance abuse), psychiatric and other relevant medical and social information. Substance use during the previous 6 months was assessed using a timeline follow-back interview (23). Body mass index (BMI) was determined upon admission (day 1). During detoxification treatment the severity of CWS was measured by a modified version of the Marijuana Withdrawal Checklist (MWC) (9, 24) and the Clinical Global Impression scale-Severity of Illness (CGI-S) (25). In its original version, the MWC consists of 10 symptoms (craving for cannabis, irritability, nervousness/anxiety, restlessness/tension, depression, anger/aggression, sleeplessness, strange dreams, loss of appetite, headache) which are rated on a 4-point scale (0 = not at all, 1 = mild, 2 = moderate, 3 = heavy) (25). Consistent with prior investigation carried out by our group (3, 9) two more clinically relevant and validated (2, 7, 10) symptoms (sweating and nausea) were added to the original MWC. The MWC was administered as a face-to-face interview by UB or by other trained physicians.

The MWC, CGI-S, and urine drug testing (see below) were performed at admission, on the next day (day 2) and subsequently every other second day until the end of the inpatient treatment period. Potential relapses were assessed during medical reviews with the help of breathalyzer and random urine drug screens (see “Exclusion Criteria”).

### Urine Cannabinoid-Analysis and Related Ratios

In clinical practice, urinary immunoassays (IA) provide immediate confirmation and detection of reported and unreported drug use, respectively (11, 14). Commercially available cannabis IA show good specificity for cannabinoids with minimal false-positive cross-reactivity from other substances (11). For this investigation, we utilized a convenient semiquantitative POCT instrument, the DRI® Cannabinoid Assay (“DRI®” (15), analyzed by a Beckmann-Coulter AU 400 chemistry analyzer.

DRI® identifies the following cannabinoids: THC, THC-OH, THC-COOH, 11-OH-delta-8-THC-COOH, 8-beta-OH-delta-9-THC, 8-beta-11-OH-delta-9-THC, and cannabidiol. DRI® provides a cannabinoid measurement range between 0 and 200 ng/ml if the analyzer is calibrated using the 200 ng/ml THC-COOH calibrator (15). Using Dri® Drugs of Abuse Immunoassays for urine screening, a sensitivity and a specificity of 91 and 96%, respectively, were observed for the detection of cannabinoids (THC-COOH, Assay cut off 4 IA-units) (26). Using a 50-ng/ml THC-COOH cutoff calibration, DRI® has demonstrated 100% accuracy verified by GC-MS with a 15 ng/ml cutoff (15). We therefore used the 200-ng/ml calibrator recommended by the manufacturer and a 50-ng/ml cutoff. For simplicity, the urine cannabinoid levels as measured by DRI® were here reported as THC-COOH levels (see also below in the “Discussion—Limitations” section). To account for the role of body fat in storage and multicompartmental pharmacokinetic redistribution of the lipophilic THC molecule and metabolites (11, 12), serial THC-COOH concentrations were adjusted for BMI (B-N-THC-COOH) and reported as nanograms per milliliter THC-COOH per kilogram square meter. To adjust for dilution or concentration of urine specimens (11), we furthermore calculated creatinine-normalized THC-COOH concentrations (C-N-THC-COOH) by dividing all serial THC-COOH concentrations by the urine creatinine concentration (g/L) with results reported in nanograms THC-COOH per milligram of creatinine (16). Creatinine levels were determined by IA from the same urine sample assayed for cannabinoids, and any sample with a creatinine concentration <20 mg/dl was considered to be adulterated (11).

### Statistics

For the intention-to-treat (ITT) analysis, we used descriptive statistics. Repeated Measures Correlation (rmcorr) for the estimation of correlations between two measures being recorded at multiple time points (27) was used to analyze the relationship between urine THC-COOH levels (or B-N-THC-COOH or C-N-THC-COOH ratios) and MWC scores across the study. Furthermore, we investigated the relationship between day-to-day change in urinary THC-COOH (delta THC-COOH) and MWC-scores (see Supplementary Material). For the investigation of influences on the temporal course of these measurements, we used Multilevel Linear Models (MLM) (28). The following control variables (possible confounders) were included in the model: age, gender, age at index cannabis use, daily amount and duration of cannabis inhalation prior to admission, prior amounts of daily cigarettes, prior psychiatric comorbidity (yes or no), and in addition, the daily gabapentin dose (22). In the MLM framework, we observed multiple measurements (level 1) for multiple participants (level 2) (28).

We also performed subgroup analyses as well based on whether patients presented with low (2–11 points) or high (12–21 points) MWC scores at admission.

With ongoing attrition of patients no longer suffering from significant CWS and thus leaving treatment, we noted an inflection point at day 16 with significant increases in average CWS (as well as THC-COOH levels) seen among patients remaining in treatment at that point (Figure 2; Supplementary Figure 2). As such, we chose to stratify the sample into “early” vs. “late” discharge subgroups based on when their discharge occurred in relationship to that mark. Comparisons between early and late discharged patients were carried out using Welch's *t* tests and χ^2^ tests as well as MLM regression analysis (see Supplementary Material).

For all tests, a significance criterion of *p* < 0.05 was used. We used IBM SPSS Statistics 21 and R (29) for our analyses, with the R package nlme (30) for MLMs. Correlation coefficients (Pearson's *r*) were defined as small (*r* > 0.1), medium (*r* > 0.2), and large (*r* > 0.3) (31).

## Results

### Sample

During the 6-year study period, 2017 detoxification treatments were carried out on the ward, with 735 of these admissions characterized by cannabinoid positivity on initial urine screen and 97 of these meeting the inclusion criteria. Eight of these however were repeat admissions of the same individual (doublets) and 11 patients declined participation, yielding a study population of 78 patients (all white) being included in the ITT analysis. Sociodemographic and clinical variables are shown in Table 1. Mean (SD) BMI was not significantly different between females [22.4 (2.93)] and males [22.5 (2.99); *t*_(24.44)_ = −0.136, *p* = 0.893]. Psychiatric comorbidity histories included borderline personality disorder, major depression, panic disorder, insomnia, and ADHD. Somatic comorbidities included allergic bronchial asthma, gastro-esophageal reflux disease, ulcerative colitis, and arthropathies not otherwise specified. Of these, none worsened during the detoxification treatment to an extent requiring further treatment.

**Table 1:** Sociodemographic and clinical variables of the ITT sample of cannabis-dependent subjects admitted for an inpatient detoxification treatment.

**Study population**	***N***	**%**	**Median**	**Mean (SD)**	**Min**.	**Max**.
Age (years old)	78	100	24	26.4 (7.0)	18	51
Females	18/78	23				
BMI (kg/m^2^)	73	93.6	22.4	22.5 (3.0)	16.5	29.5
Age (years) at first-ever cannabis use	73	93.6	17	18.0 (4.8)	9	33
Duration (years) of cannabis use prior to admission	73	93.6	8	9.4 (2.2)	0.25	36
Daily amount (g) of cannabis inhalation during the 6 weeks prior to admission	73	93.6	2	2.2 (1.5)	0.5	10
(Tobacco) cigarettes per day	72	92.3	20	18.6 (7.8)	0^a^	40
Patients requiring PRN medication	44	56.4				
Patients without educational qualifications	04	5.1				
Patients with primary school education	31	39.7				
Patients with secondary school education	18	23.1				
Patients with general university entrance certificate (Abitur)	05	6.4				
Patients having completed vocational training	26	33.3				
Unemployed patients	38	48.7				
Patients with a history of psychiatric comorbidity	33	42.3				
Patients with a history of somatic comorbidity	4	5.1				

a *Three patients reported nil use of nicotine*.

### Treatment Durations and Attrition Rate

**Figure 1:**
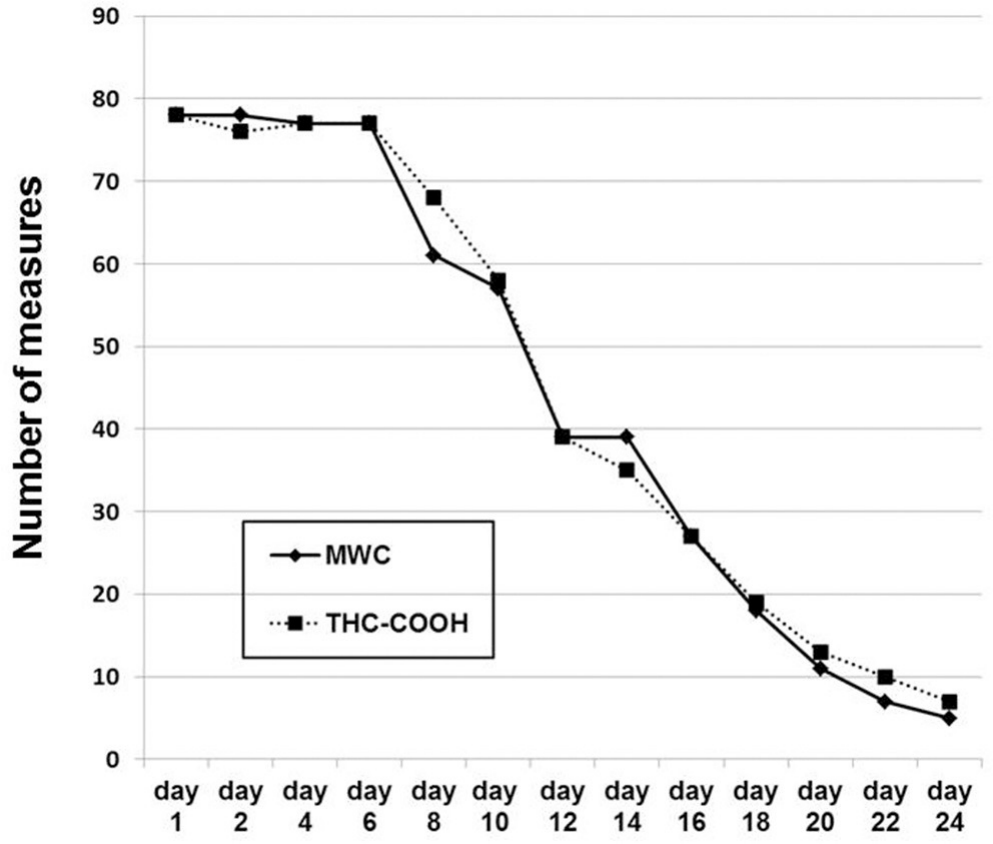
The number of serial investigations across the study. The number of patients remaining in the study is equivalent to the maximal number of any measure at any given day. Day 1, day of admission. At every study day, MWC was always linked to a CGI-S measurement.

Nine (11.5%) participants dropped out of the study. All of these patients discontinued inpatient treatment prematurely, within days 4–13; remaining patients (*n* = 69, 88.5%) underwent planned discharge. The attrition rate (dropouts plus regularly discharged patients) is illustrated in Figure 1. The mean (SD) inpatient detoxification lasted 14.6 days (6.5), with a median of 14 days, and minimum of 6 and maximum of 24 days.

### PRN Medication

See Supplementary Material.

### Postdischarge Treatment

Most treatment completers (*n* = 37, 47.4% of all patients) were referred to an outpatient program at the same clinic (32) while 21 patients (26.9%) were referred to a specialized long-term rehabilitation facility.

### Course of Measured Variables

**Figure 2:**
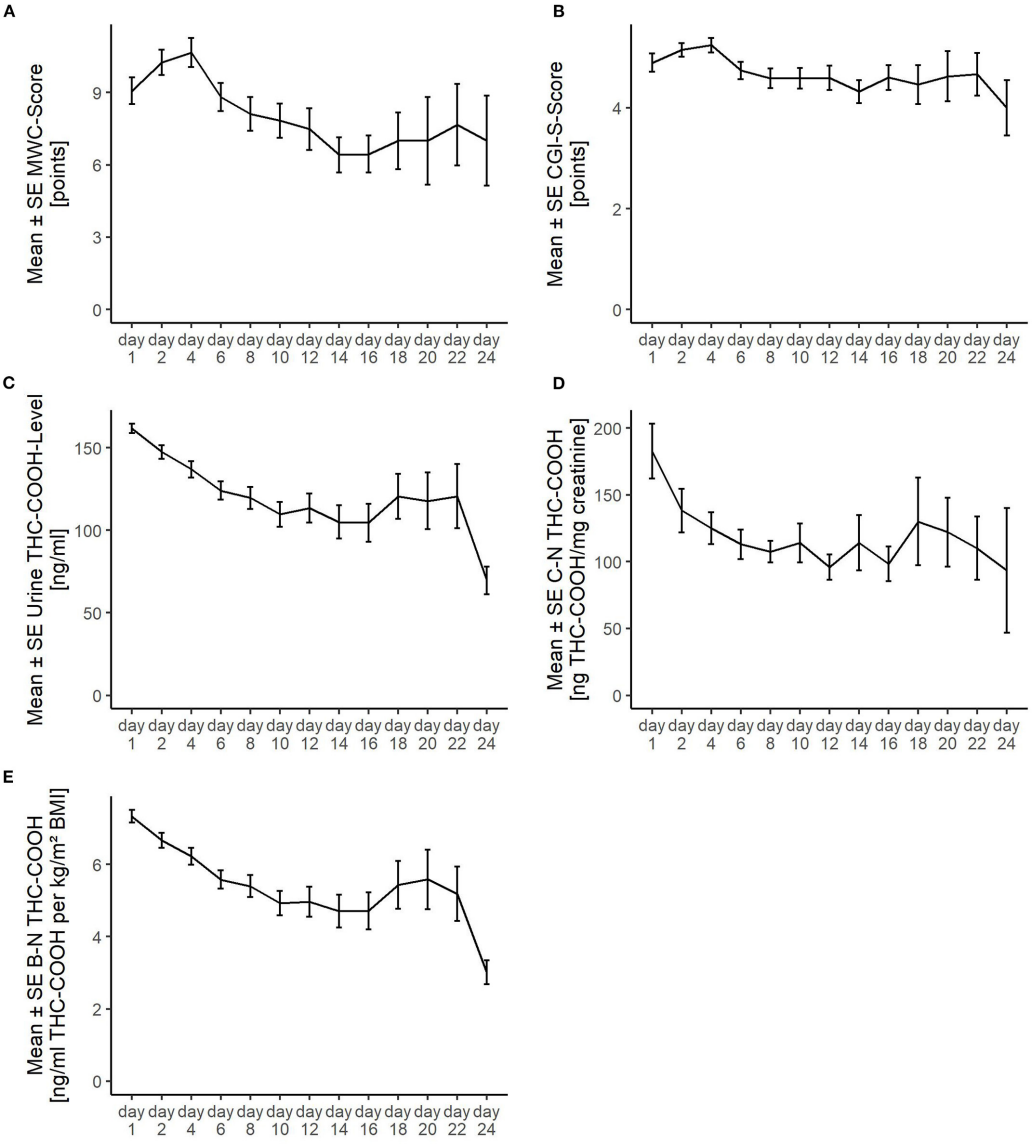
Course of the detoxification treatment as measured **(A)** via the Marijuana Withdrawal Checklist (MWC); **(B)** Clinical Global Impression-Severity (CGI-S); **(C)** levels of urinary 11-nor-delta-9-tetrahydrocannabinol-9-carboxylic acid (Urine THC-COOH); **(D)** creatinine-normalized THC-COOH (C-N-THC-COOH); and **(E)** BMI-normalized THC-COOH (B-N-THC-COOH). SE, standard error. The striking transient deterioration between days 16 and 22 correlated with the removal of a “dilution effect” exerted by improved patients leaving treatment prior to day 16.

Figure 1 shows the number of the serial MWC ratings and urinary THC-COOH measurements across the study. Their decreasing numbers over time reflect the nine dropouts and regular discharge of patients as outlined above in Figure 1. Figure 2 shows the values of the measurements across the study. The striking transient deterioration between days 16 and 22 correlated with the removal of a “dilution effect” exerted by improved patients leaving treatment prior to day 16.

### Relationship Between CWS and Urine THC-COOH Across the Study

Using *rmcorr*, which analyzes the relationship between MWC and the respective metabolite ratios at every point of measurement, positive correlation was identified between MWC and THC-COOH (*r* = 0.248 [0.152, 0.339], *df* = 388, *p* < 0.001). A significant positive correlation was also found between MWC and B-N-THC-COOH (*r* = 0.249 [0.154, 0.341], *df* = 388, *p* < 0.001). The association between MWC and C-N-THC-COOH was not significant (*r* = 0.096 [−0.004, 0.195], *df* = 382, *p* = 0.059).

To account for possible confounding variables (listed above in the “Statistics” section), we used MLM. The random intercept and random slopes models were in all cases superior to the intercept-only and random intercept-only models (see “Statistics”). The chosen random intercept and slopes models' calculations provided the following results: a significant association was confirmed between MWC scores and THC-COOH ratios (*b* = 0.026 [0.014, 0.037], *p* < 0.001). The models also demonstrated a significant positive association between MWC scores and B-N-THC-COOH (*b* = 0.572 [0.338, 0.805], *p* < 0.001). Finally, the relationship between MWC scores and C-N-THC-COOH ratios also emerged as significant (*b* = 0.005 [0.0002, 0.009], *p* = 0.040), with the daily gabapentin dose being the decisive factor, *b* = 0.001 [0.0005, 0.002].

### Influence of Admission (Baseline) CWS Severity

For those patients (*n* = 21) who presented with high CWS severity (12–21 MWC points) upon admission, C-N-THC-COOH values significantly correlated with the MWC scores across the whole study (*r* = 0.247 [95% CI, 0.094 to 0.39]; *p* = 0.002). This association remained significant after incorporating the control variables into the model (*b* = 0.011 [95% CI, 0.003 to 0.019]; *p* = 0.006). Conversely, for those 46 subjects who presented at admission with low CWS severity (2–11 MWC points), no significant correlation was identified with C-N-THC-COOH ratios across the whole study (*r* = −0.043 [95% CI, −0.173 to 0.088]; *p* = 0.519; *b* = 0.0004 [95% CI, −0.005 to 0.005]; *p* = 0.891).

### Predictors of CWS and Urine THC-COOH Values (as well as the Ratios)

To further clarify the above-described associations, we analyzed time course and a range of remaining control variables (see “Methods—Statistics”) as possible predictive factors influencing MWC scores, urine THC-COOH levels, C-N-THC-COOH, and B-N-THC-COOH ratios. Again, the random intercept and random slopes models were in all cases superior to the other models of MLM; the respective results of these models are presented below.

### MWC Scores

MLM revealed the factor “time” to be significant (*p* = 0.009) with a negative regression coefficient (*b*) of −1.078 [95% CI, −1.873 to −0.284]. In other words, the MWC score decreased 1.08 points on average from measurement to measurement when all control variables were held constant. The other significant association noted was that between MWC scores and Gabapentin dose (*b* = 0.001 [95% CI, 0.0006 to 0.002]; *p* = 0.003), i.e., the higher the daily gabapentin dose, the higher the MWC scores. The remaining variables did not significantly predict the MWC score.

### Urine THC-COOH Levels

Time was identified as a significant factor (*p* = 0.003) in predicting urine THC-COOH levels with a negative regression weight (*b*) of −13.562 [95% CI, −22.492 to −4.632]. In other words, with remaining variables being held constant, THC-COOH ratios decreased from measurement to measurement by over 13 points on average. Age at first-ever cannabis use also emerged as a significant predictor (*b* = −3.391 [95% CI, −6.491 to −0.291]; *p* = 0.035) with higher THC-COOH ratios for those with an earlier onset of cannabis use. The interaction between time and gender was significant as well (*b* = −4.549 [95% CI, −8.721 to −0.377]; *p* = 0.035), indicating a faster decline of THC-COOH levels in male patients.

### C-N-THC-COOH Ratios

For all urine samples, creatinine was >20 mg/dl. Neither “time” nor the other control variables influenced the C-N-THC-COOH ratios significantly.

### B-N-THC-COOH Ratios

The factor “time” was significant (*p* < 0.001) with a negative regression weight (*b* = −0.731 [95% CI, −1.154 to −0.308]). In other words, with the other variables held constant, B-N-THC-COOH ratios decreased from measurement to measurement by 0.73 points on average. The interaction between time and gender was also significant (*b* = −0.219 [95% CI, −0.417 to −0.020]; *p* = 0.033), indicating a faster decline of B-N-THC-COOH levels in males. The remaining control variables did not influence the B-N-THC-COOH ratios significantly.

### Using the Day-to-Day Change in Urinary THC-COOH Levels (Delta THC-COOH-Levels) Instead of the Absolute THC-COOH Levels

All aforementioned significant relationships and influences identified by using the absolute urinary THC-COOH levels as outcome variable were confirmed by substituting these by using the relative delta THC-COOH levels (Supplementary Figure 1). The other results were also not altered as the relationships and influences remained insignificant (see Supplementary Material).

### Relationship Between MWC and CGI-S Scores

Using rmcorr, a significant correlation between MWC and CGI-S scores was identified (*r* = 0.812 [95% CI, 0.776 to 0.843]; *p* < 0.001). Using MLM to adjust for the influence of control variables, the relationship between MWC and CGI-S scores remained significant, with *b* = 3.310 [95% CI, 2.972 to 3.652], and *p* < 0.001.

### Early- vs. Late-Discharged Patients

See Supplementary Material.

## Discussion

### Serial Positive Correlation of Urine THC-COOH and CWS Across the Study and Factors Influencing Their Slopes

Individuals seeking inpatient detoxification for their CDS—without other significant comorbidities/coexisting substance use disorders—are relatively rare in our experience, as reflected in the prolonged recruitment and study period of 6 years. To the best of our knowledge, this study is the first to demonstrate the significant association between urine THC-COOH levels and CWS severity, which proved to be a robust finding via regression analyses, rmcorr and MLM. The strength of the association however was moderate at best (*r* = 0.248) and disappeared when evaluating creatinine-adjusted cannabinoid levels (*r* = 0.096, *p* = 0.059). A significant positive correlation however between these variables (C-N-THC-COOH and MWC) was restored if PRN medication use was left in the model. As we have also found that the PRN medication significantly predicted the CWS severity, it was likely that a significant correlation between MWC scores and C-N-THC-COOH ratios may be observed in patients with high CWS levels only. Indeed, this was the case in patients with MWC scores >11 points at admission, again with moderate effect size (*r* = 0.247).

### The Controversial Role of Creatinine-Adjusted Drug Screens in Routine Practice

To reduce the influence of urine dilution upon measured drug and metabolite values, creatinine adjustment or normalization is recommended as the scientific standard, including the assaying of urine THC levels (11). However, such adjustment is not generally carried out in routine practice, which typically relies only on threshold or cutoff measurement/detection (33). The reliability of the urine creatinine level as a “dilution marker” is also limited by the effects of protein concentration in the diet, muscle mass, physical activity and even emotional stress, and urine creatinine level may accordingly vary greatly throughout the day (34, 35). As such, it must be understood that urine creatinine level at any given point in time comprises a single “snapshot.”

One could speculate that PRN medication altered urine concentration by an effect on GFR or solute reabsorption. Neither mechanism however has been shown at therapeutic doses of gabapentin or chlorprothixene to the best of our knowledge nor has an additive osmolar effect (more solute, i.e., gabapentin or chlorprothixene in the urine).

### Gender Effect

Urine THC-COOH levels decreased significantly faster in males, while females remained longer in the study (i.e., >16 days), suggesting a protracted withdrawal in females. This is consistent with previous studies showing worsened CWS levels in females vs. males (7, 9).

### BMI and Other Factors Putatively Influencing Both CWS- and Urinary THC-COOH Slopes

The rapid decline in urinary THC-COOH levels in males appeared to be independent from BMI normalization, and this may argue against a prolonged redistribution phase of THC from adipose tissue deposits (13, 14) as the key factor for delayed urine elimination of THC-COOH (12, 13). Similarly, no correlation between BMI and plasma THC levels in chronic cannabis smokers during 7 abstinent days was identified (36).

Female and male BMI levels were not significantly different in this study population, which may suggest that the suggested gender-specific differences in both THC elimination and protracted CWS may not necessarily be the consequence of body composition differences. Conversely, one could argue that BMI may not be the best marker to identify the individual fat proportion and distribution. In order to better characterize the potential role of adiposity on cannabinoid metabolism and elimination, alternate methods such as skin caliper testing, Dual Energy X-ray Absorptiometry (DEXA), or Magnetic Resonance Imaging (MRI) might be considered (37).

Neither age, amount, or duration of prior cannabis intake nor history of comorbidities were shown to influence MWC, THC-COOH, or their interaction in this study. Furthermore, although age at initial cannabis use predicted urine THC-COOH levels, it did not predict either CWS severity or the positive association between THC-COOH and MWC.

### PRN Medication Effectiveness

See Supplementary Material.

### Replacing the Absolute THC-COOH Levels With the Day-to-Day Change in THC-COOH Levels

Using each day's absolute THC-COOH level (Figure 2C) is not the same as day-to-day change in level (Supplementary Figure 1). It seems plausible that it is the degree of THC-COOH decline itself, not the absolute level, that indirectly drives withdrawal [provided that the THC-COOH decline is closely related with the THC decline which pharmacologically drives withdrawal (3, 11, 13)]. However, we found no relevant differences regarding our results when replacing the absolute THC-COOH levels with the (relative) day-to-day change in urinary THC-COOH levels as an outcome variable (see Supplementary Material). In this context, it should be emphasized that all patients reported that their last cannabis use had taken place within the last 24 h before admission (according to our inclusion criteria). Thus, this time span seems to be appropriate for our accuracy purposes.

### Protracted CWS and THC-COOH Elimination

Most of the study population (e.g., the “early” group; *n* = 58 including dropouts) had been discharged normally before day 16 due to sufficient clinical improvement levels. Conversely, the findings of the “late” group (*n* = 20), presenting with both considerable CWS intensity and urine THC-COOH levels, may indicate the existence of a distinct subset of THC users—around 30% in our sample—characterized by a protracted withdrawal course (38–40). The symptom patterns and trajectories of these two groups are consistent with those of the previously postulated CWS subtypes A and B (3), respectively. It would be worth investigating whether genetic variations of cannabis-metabolizing enzymes account for these subtypes' differences (13, 41).

In our late-discharged group, the average THC-COOH values did not drop below the cutoff value of 50 ng/ml even after 24 abstinent days. While this is in line with previous findings (42, 43), it also supports the existence of a special population among chronic cannabis users with a delayed THC terminal-phase elimination from the body (16, 43–46). While prolonged CWS courses have previously been described and ascribed to psychiatric comorbidities (39, 40), our late group showed no such association with psychiatric comorbidity nor with age nor cannabis history data. What we did find was a disproportionate female preponderance within the late group, with increased urine THC-COOH levels, but not with MWC scores. These observations are consistent with previous observations of females experiencing a more complicated CWS than males (3, 7). An alternative explanation would be that the patients in the late group had consumed cannabis during the study period; the likelihood of such occurrence was however here minimized by the measures utilized to detect possible hidden drug and alcohol use.

### Serial Positive Correlations Between MWC and CGI-S

The present study confirmed our previous findings that the MWC score is a valuable predictor of the disease burden experienced by patients with CDS abstaining from use, as measured via the CGI-S (9). Notably, in patients with a protracted CWS (*n* = 20, 27.4%), the average CGI-S score did not drop over time below the 4-point mark, indicating: “…overt symptoms causing noticeable but functional impairment or distress…” (25). This demonstrates a persistent illness burden in a subgroup of heavy/long-term cannabis users despite detoxification, who may require more intensive postdetox rehabilitation (3). It could be argued that durable executive and social deficits (47, 48) were the main factors behind the functional impairment of this (primarily female) group demonstrating slower elimination of cannabinoids and protracted CWS, and these phenomena might be amplified by potential gender differences in the regulation of the brain endocannabinoid system (7). As a side note, this constellation of pharmacokinetic and pharmacodynamic differences might also explain the observation that females seem to progress more rapidly from first regular cannabis use to cannabis dependence than males (7).

Cognitive functions of this group may also have been affected by residual plasma THC [as with more recently abstinent chronic cannabis users (3, 44)], but this hypothesis is not consistent with recent findings (49).

### Strengths of the Study

This study provides insight into the course of CWS among treatment-seeking adult CDS patients during detoxification, and is the first to investigate the feasibility of utilizing a simple urine-based POCT to prognosticate about the likely severity of CWS. The sample was relatively large for this type of study, allowing for adequate study power to investigate not only routine variables such as gender, but also less frequently considered variables including BMI, urine creatinine concentration, etc.

### Limitations of the Study

Immunoassays are well known to yield false-positive results (15), and for optimal specificity, more accurate GC-MS assays should be employed (11, 13). The point of this study however was to investigate the potential of a convenient, point-of-care, semiquantitative IA for use under routine inpatient conditions. Inherent in the methodology therefore is the risk that the cannabinoids identified measured by the DRI® test may not have exclusively comprised THC-COOH. Nonetheless, this main THC metabolite increases in the urine within a few hours after cannabis use, with specificity/accuracy increasing over time, paralleling the number of abstinent days (11, 12, 49). Although one could also hypothesize patients' intentional use of adulterants to produce false-negative urine screens (50), all patients sought treatment of their own accord and presented with a high degree of motivation, lessening the likelihood of such deception.

A ratio of 1.5 (21) for comparison of later-to-earlier C-N-THC-COOH levels was used here as evidence of hidden/undisclosed cannabis relapse. Although this value has been criticized because of its potential low sensitivity, the utilization of a lower ratio of 0.5 (13, 44) did not alter our results (data not shown here).

Consistent with other studies focusing on gender effect on CWS severity (7), we did not control for female participants' menstrual period phase, which may have influenced CWS severity levels.

Roughly half of the patients had been discharged by day 12, which lowers the statistical power over the second half of the study. However, the utilization of both rmcorr and MLM regression analyses should reduce inaccuracies and biases associated with that attrition (27, 28); see also Supplementary Material—Methods.

Our results are rather specific for detoxification units in our country which, for cannabis is usually performed in psychiatric hospitals with similar personnel and material structures. However, for the general population of cannabis users, our results may not be representative.

We did not perform a direct analysis of the association between urine cannabinoid levels (by POCT) and the severity of CDS or cannabis use disorder (CUD) which should comprise a future project. It would also be interesting to expand this investigation to the outpatient treatment arena, where environmental stress and CWS might be more intense and prolonged, and might yield a greater association between urine cannabinoid levels and CWS than the moderate one demonstrated in this inpatient study. In this context, a more detailed investigation of the change of the co-use of daily nicotine alongside further studies to these issues would be particularly informative as concurrent tobacco use and possible tobacco withdrawal may modulate the severity of CWS, CDS, and cannabis use (1, 3, 11). Ignoring concurrent tobacco use is a major limitation of the present study. The same applies as for the fact that we did not determine the exact time of the last cannabis use of the participants, which, however was within the last 24 h before admission (see inclusion criteria in the “Methods” section and the section further above where the results after replacing the absolute THC-COOH-levels with the day-to-day change in THC-COOH levels are discussed). Self-reported time of last cannabis use has some limitations. However, using this variable with more direct pharmacological relationship to the outcome variable (CWS), even though it has some measurement limitations, might be a sophisticated alternative to using an objective variable with assumably weaker pharmacological relationship to the CWS as we did with defining the date of admission as temporal baseline. As we used self-reports to inform about remote and recent drug history, reporting or recall bias might have also influenced our results. To mitigate this bias for the CWS rating, we performed MWC face to face as well as the CGI-S.

## Conclusions

Throughout this 24-day study, the urine THC-COOH levels significantly predicted the severity of CWS, as measured by the MWC. After creatinine adjustment, serial THC-COOH values significantly correlated with serial MWC scores only in those subjects with high MWC scores (>11 points) at admission. The correlation levels were generally moderate (*r* ~ 0.25). Female gender correlated significantly with both a delayed decrease in urine cannabinoid levels and with prolonged CWS. According to the CGI-S, these CWS levels were characterized by significant illness severity, which is consistent with a previously postulated “nonpeaking” CWS-subtype B (3). Conversely, those patients with a nonprotracted CWS showed the typical “peaking” character of the CWS-subtype A (3), which is more commonly seen and reported. The levels of MWC and CGI-S were here strongly correlated (*r* = 0.81), suggesting that the CDS disease burden is comparable with that of other medical conditions.

## Data Availability Statement

The raw data supporting the conclusions of this article will be made available by the authors, without undue reservation.

## Ethics Statement

The studies involving human participants were reviewed and approved by Ethics commitee of the Medical Faculty of the University of Duisburg-Essen. The patients/participants provided their written informed consent to participate in this study.

## Author Contributions

UB: conception, design, data collection, and drafting the article. BC and MS: analysis of the data. All authors interpretation of data and revising the manuscript critically for important intellectual content.

## Conflict of Interest

NS has received honoraria for several activities (e.g., advisory board membership, lectures, manuscripts) from AbbVie, Camurus, Hexal, Janssen-Cilag, MSD, Medice, Mundipharma, Reckitt-Benckiser/Indivior, and Sanofi-Aventis. During the last 3 years he has participated in clinical trials financed by the pharmaceutical industry. The remaining authors declare that the research was conducted in the absence of any commercial or financial relationships that could be construed as a potential conflict of interest.
