# Supplementary material for: Is the Urine Cannabinoid Level Measured via a Commercial Point-of-Care Semiquantitative Immunoassay a Cannabis Withdrawal Syndrome Severity Predictor?
Source: Front Psychiatry. 2020 Dec 3;11:598150. doi: 10.3389/fpsyt.2020.598150 (PMC7744589; doi:10.3389/fpsyt.2020.598150)
Supplement: Supplementary file 1 [file Data_Sheet_1.PDF]

Supplemental Material

Is the urine cannabinoid level measured via a commercial point-of-care semi-quantitative immunoassay a cannabis withdrawal syndrome severity predictor?

Benedikt Bernd Claus,[1] Michael Specka,[2] Heath McAnally,[3] Norbert Scherbaum,[2] Fabrizio Schifano[4], Udo Bonnet[1,2;*]

[1] Department of Psychiatry, Psychotherapy and Psychosomatic Medicine, Evangelisches Krankenhaus Castrop-Rauxel, Academic Teaching Hospital of the University of Duisburg-Essen, D-44577 Castrop-Rauxel, Germany

[2] LVR-Hospital Essen, Department of Psychiatry and Psychotherapy, Faculty of Medicine, University of Duisburg-Essen, D-45147 Essen, Germany

[3] University of Washington School of Medicine, Department of Anesthesiology and Pain Medicine, Seattle, WA, USA

[4] University of Hertfordshire, *Psychopharmacology, Drug Misuse and Novel Psychoactive Substances Research Unit;* School of Life and Medical Sciences; Hatfield, Herts (UK)

*Corresponding author at: Department of Psychiatry, Psychotherapy and Psychosomatic Medicine, Evangelisches Krankenhaus Castrop-Rauxel, Grutholzallee 21, D-44577 Castrop-Rauxel, Germany. E-mail address: udo.bonnet@uni-due.de (Prof. Dr. med. Udo Bonnet).

Methods

Statistics

Advantages of rmcorr and MLM

The Repeated Measures Correlation (*rmcorr*) is an ideal procedure to investigate the relationship of two variables measured at multiple/serial time points as every data point can be included in the analysis. This yields greater statistical power without the risk of drawing false conclusions from averaging multiple values into one and computing just one standard correlation coefficient. The *rmcorr* considers the relationship per individual participant and averages this relationship over many participants [27]. This is quite similar to the idea of Multilevel Linear Models (*MLM*) [28], in which data points can be grouped in higher levels, such as longitudinal data points in participant levels [28]. Conversely, the conventionally used repeated measures *ANOVA* relies on listwise case deletion, thus excluding a participant from an analysis because of as few as one missing data point. This does not apply to *MLM*, which, again, models the relationship per individual participant and subsequently averages this relationship over every participant [28]. This may result as well in considerably more statistical power in the analysis.

Comparisons between early and late discharged patients

With ongoing attrition of patients no longer suffering from significant CWS and thus leaving treatment, we noted an inflection point at day 16 with significant increases in average CWS (as well as THC-COOH levels) seen among patients remaining in treatment at that point (Figure 2 and Supplemental Figure 2). As such, we chose to stratify the sample into “early” vs. “late” discharge subgroups based on when their discharge occurred in relationship to that mark. Comparisons between early and late discharged patients were carried out using Welch’s *t*-tests and χ^2 tests.

To further investigate the differences between these subgroups, *MLM* [28] was used to analyze differences in MWC scores as well as THC-COOH ratios according to both “time” and discharge subgroup status. In this framework, three models could be estimated: (i) the intercept-only model, (ii) the random intercept-only model and (iii) the

random intercept and random slopes model. To find the best model among these three, we used ANOVA.

Results

PRN medication

Forty-five (57.7%) patients required PRN medication to treat withdrawal symptoms, i.e. gabapentin (*n* = 41) or chlorprothixene (*n* = 4). A total of 360,400mg of gabapentin [22] was administered during 400 study days to 45 patients, averaging 900 mg per day. These patients stayed a cumulative 660 days in our inpatient treatment. The total sample of 78 patients stayed a cumulative 1067 days.

Using the day-to-day change in urinary THC-COOH levels (delta THC-COOH-levels) instead of the absolute urine THC-COOH-levels

The course of the mean delta THC-COOH-levels is shown in Supplemental Figure 1. In the following, the relationships between CWS (MWC scores) and delta THC-COOH-levels (as well as the ratios) considering the control variables (as listed in the Statistics section) are evaluated. Using MLM, the random intercept and random slopes models were in all cases superior to the intercept-only and random intercept-only models (see Statistics). The chosen random intercept and slopes models' calculations provided the following results: a significant association was also found between MWC scores and delta THC-COOH-levels across the study (b = 0.024 [0.013, 0.035], $p < 0.001$). The models again demonstrated a significant positive association between MWC scores and delta B-N-THC-COOH (b = 0.571 [0.333, 0.808], $p < 0.001$). The relationship between MWC scores and delta C-N-THC-COOH also emerged as significant (b = 0.005 [0.0003, 0.009], p = 0.037).

Influence of admission (baseline) CWS severity

For those patients (n = 21) who presented with high CWS severity (12-21 MWC points) upon admission, delta C-N-THC-COOH values significantly correlated with the MWC scores across the whole study (r = 0.247 [95% CI: 0.094 to 0.39], p = 0.002). This association remained significant after incorporating the control variables into the model (b = 0.012 [95% CI: 0.002 to 0.021], p = .016). Conversely, for those 46 subjects who presented at admission with low CWS severity (2–11 MWC points), no significant

correlation was identified with delta C-N-THC-COOH ratios across the whole study (r = -0.043 [95% CI: -0.173 to 0.088]; p = .519; b = -0.0005 [95% CI: -0.004 to 0.004], p = 0.826).

Predictors of urine delta THC-COOH values (as well as the ratios)

Urine delta THC-COOH-levels

Time was identified as a significant factor (p < .001) in predicting urine delta THC-COOH levels with a negative regression weight (b) of -14.95 [95% CI: -22.492 to -4.632]). Age at first-ever cannabis use also emerged as a significant predictor (b = -3.671 [95% CI: -6.771 to -0.570], p = 0.023). The interaction between time and gender was significant as well (b = -4.796 [95% CI: -9.070 to -0.522], p = 0.030), indicating a faster decline of delta THC-COOH levels in male patients.

Delta C-N-THC-COOH-ratios

Neither “time” nor the other control variables influenced the delta C-N-THC-COOH ratios significantly.

Delta B-N-THC-COOH-ratios

The factor “time” was significant (p < 0.001) with a negative regression weight (b = -0.789 [95% CI: -1.120 to -0.376]). Furthermore, we found a significant influence of age at first-ever cannabis use (b = -0-202 [95% CI: -0.375 to -0.0029], p = 0.025). The interaction between “time” and gender was also significant (b = -0.238 [95% CI: -0.441 to -0.036], p = 0.023), indicating a faster decline of B-N-THC-COOH levels in males. The remaining control variables did not influence the delta B-N-THC-COOH ratios significantly.

Early- versus late-discharged patients

Figure 1 shows that the number of inpatients decreased steadily over time, with a 50% attrition rate by day 12. Figure 2 shows a transient increase in all measures beginning on day 16, corresponding to a reduced “dilution” effect conferred by those patients with lower MWC scores and THC-COOH levels leaving the study prematurely (regular discharges plus drop-outs). By day 14, this difference was significant for both MWC (t = -4.35, df = 28.98, p < 0.001) and CGI-S scores (t = -4.48, df = 23.89, p 0<.001), but not for THC-COOH values (t = 0.94, df = 9.77, p = 0.372). To analyze the predictors of

this higher acuity “late” group we divided the study population into two subgroups; the “early” discharged patients, i.e., all those who had left the study by day 16 (n = 58, which included all dropouts) and the “late” discharged patients (n = 20) still remaining in the study thereafter. The trajectories of these “early” and “late” discharged patients are demonstrated in Supplemental Figure 2. MWC scores (b = 0.33 [95% CI: 0.03 to 0.63], p = 0.031), CGI-S scores (b = 0.15 [95% CI: 0.08 to 0.23], p < 0.001) and urine THC-COOH levels (b = 5.61 [95% CI: 2.01 to 9.21], p = 0.002) showed significantly decreased rate of decline in the “late” group.

Chi-squared tests revealed significantly more females than males in the “late group” ($\chi^2(1)$ = 4.34, p = 0.037). Neither the use of PRN medication ($\chi^2(1)$ = 0.176, p = 0.675) nor psychiatric comorbidity ($\chi^2(1)$ = .080, p = 0.777) predicted “late” group CWS trajectory, nor did other analyzed variables shown in Supplemental Table 1.

Discussion

PRN medication effectiveness

In a well-controlled proof-of-concept study gabapentin was shown to be effective in reducing CWS [22]. Not surprisingly, patients with high MWC scores more often requested PRN medication, and this association was potentially confounded by a gender effect.

Reference numbers according the main article

Supplemental Tables and Figures

Supplemental Table 1: Possible predictors of early- versus late-discharged patients

	Early discharged Patients			Late discharged patients					
Measure	*M*	*SD*	*n*	*M*	*SD*	*n*	t^a	*Df*	*p*
Age	26.40	6.89	58	26.30	7.64	20	.05	30.34	.961
BMI [kg/m^2]	22.68	2.91	53	21.91	3.06	20	.98	32.79	.334
Age (years) at first-ever cannabis use	18.53	5.18	53	16.55	3.50	20	1.87	50.74	.067
Duration (years) of cannabis use prior to admission	9.03	5.30	53	10.36	7.87	20	-.7	25.79	.490
Daily amount (g) of cannabis inhalation during the 6 weeks prior to admission	2.27	1.59	53	2.15	1.24	20	.35	44.01	.728
(tobacco) cigarettes per day	18.79	8.79	52	18.25	4.67	20	.34	62.72	.738
Gender (female/male)^b	10/48		58	8/12		20	4.34	1	.037
PRN (no/yes)^b	21/31		52	7/13		20	0.18	1	.675
Psychiatric comorbidity (yes/no)^b	34/24		58	11/9		20	0.80	1	.777

^{a}Welch's *t*-Test

$^b X^2$-tests because of count data

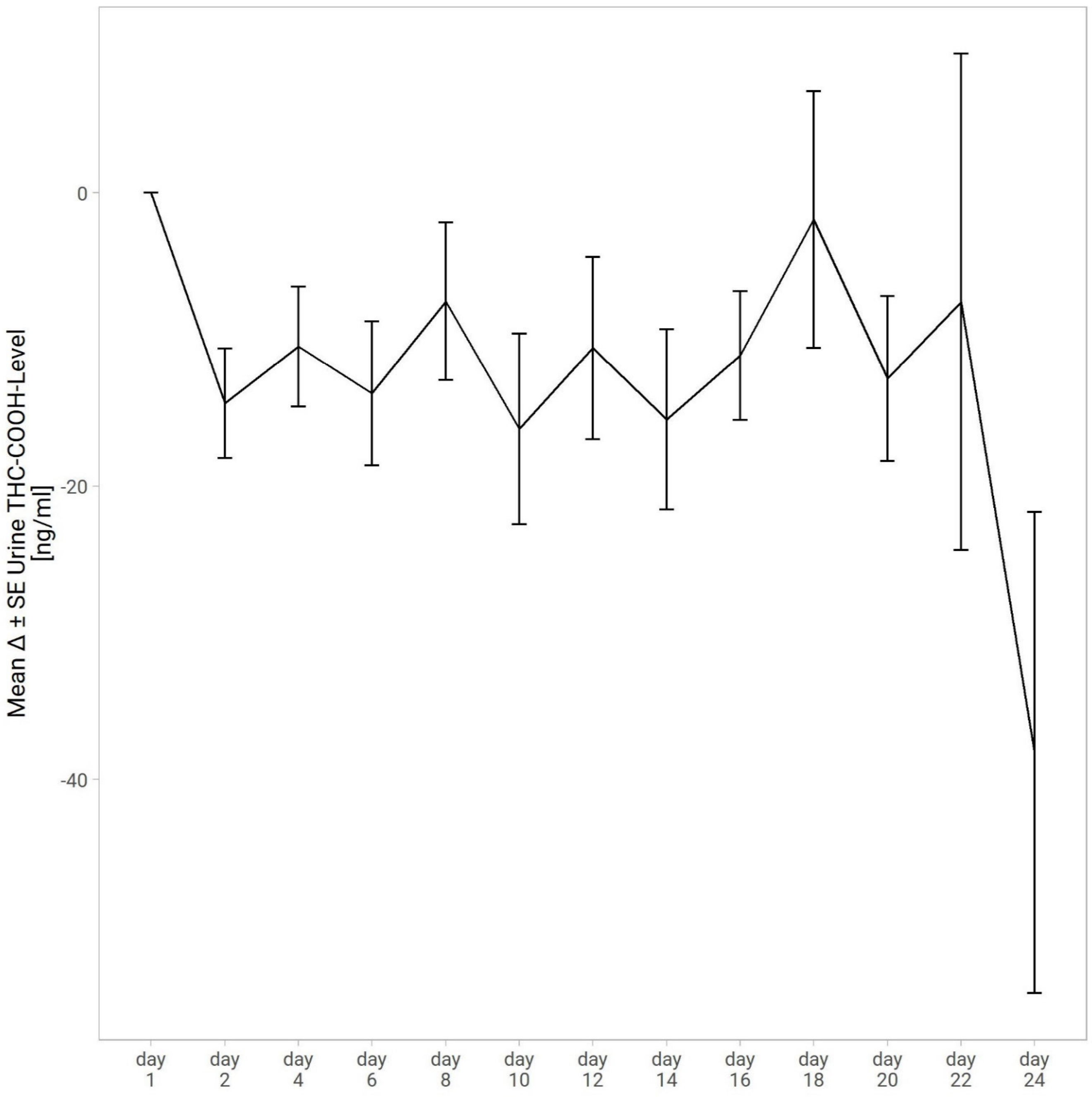

Supplemental Figure 1: Course of the mean day-to-day change scores in levels of urinary 11-nor-delta-9-tetrahydrocannabinol-9-carboxylic acid (Urine THC-COOH). SE = standard error.

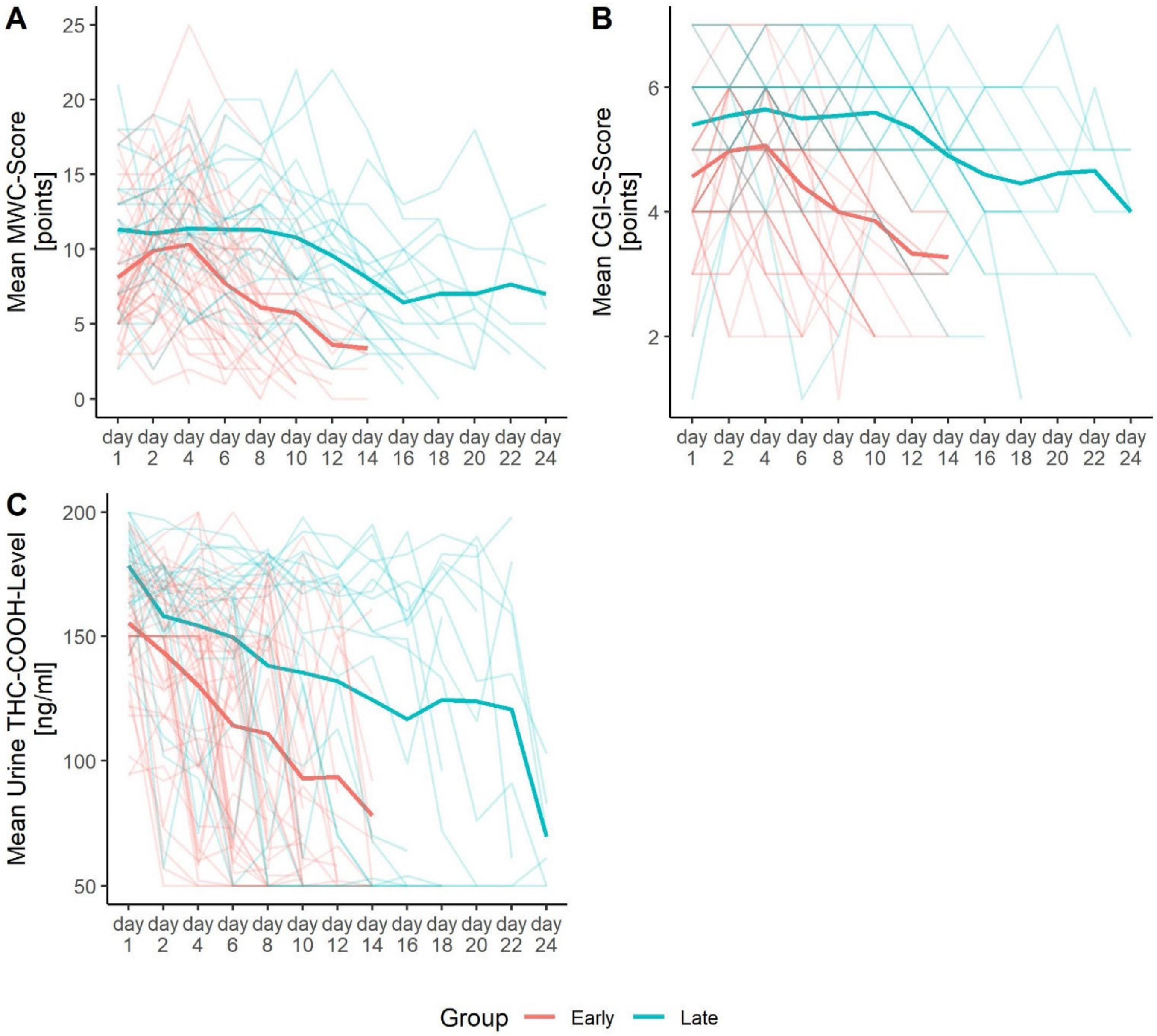

Supplemental Figure 2: Individual detoxification trajectories as measured (A) via the Marijuana Withdrawal Checklist (MWC), (B) via the Clinical Global Impression scale – Severity (CGI-S), and (C) via levels of urine 11-nor-delta-9-tetrahydrocannabinol-9-carboxylic acid (Urine THC-COOH) - split between two subgroups at treatment day 16 distinguishing between “early” (red lines) and “late” (blue lines) discharged patients. Bold lines indicate the average trajectory per subgroup. The late group had a more severe CWS (MWC score) and burden of disease (CGI-S) as well as higher urine THC-COOH levels than the early patients. These distinct withdrawal courses (with early, “peaking” CWS course vs. late and more severe, protracted, “non-peaking” CWS-course) parallel previously postulated CWS-subtype A and CWS-subtype B, respectively [3].
